# A robust approach to differentiate human monocyte-derived microglia from peripheral blood mononuclear cells

**DOI:** 10.1016/j.xpro.2022.101747

**Published:** 2022-10-05

**Authors:** Hazel Quek, Carla Cuní-López, Romal Stewart, Yi Chieh Lim, Tara L. Roberts, Anthony R. White

**Affiliations:** 1 Mental Health and Neuroscience, QIMR Berghofer Medical Research Institute, Brisbane, QLD 4006, Australia; 2 School of Biomedical Science, The University of Queensland, Brisbane, QLD 4072, Australia; 3 School of Biomedical Sciences, Queensland University of Technology, Brisbane, QLD 4059, Australia; 4 Faculty of Medicine, The University of Queensland, Brisbane, QLD 4006, Australia; 5 Brain Tumor Biology, Danish Cancer Society, Copenhagen 2100, Denmark; 6 Ingham Institute for Applied Medical Research and School of Medicine, Western Sydney University, Liverpool, NSW 2170, Australia

**Keywords:** Cell biology, Cell culture, Cell isolation, Health sciences, Immunology

## Abstract

Microglia are implicated in most neurodegenerative diseases. Here, we present a robust and efficient protocol to differentiate monocyte-derived microglia-like cells (MDMi) from whole blood. The protocol consists of three parts. The first part will describe two methods for PBMC isolation. This will be followed by MDMi differentiation, and lastly, the characterization of MDMi by immunocytochemistry. MDMi can be used to investigate microglial-related responses in various age-related neurodegenerative diseases and can be applied to drug testing on a personalized basis.

For complete details on the use and execution of this protocol, please refer to Quek et al. (2022).

## Highlights

• A robust protocol to generate microglia-like cells (MDMi) from human peripheral blood
• In-depth guide to achieve maximum PBMC cell recovery for consistent MDMi culture
• MDMi are transdifferentiated using growth factors (IL-34 and GM-CSF)
• MDMi transdifferentiated for 14 days express microglia-specific marker

## Abstract (editor highlights)

Publisher’s note: Undertaking any experimental protocol requires adherence to local institutional guidelines for laboratory safety and ethics.

## Graphical abstract

**Figure:**
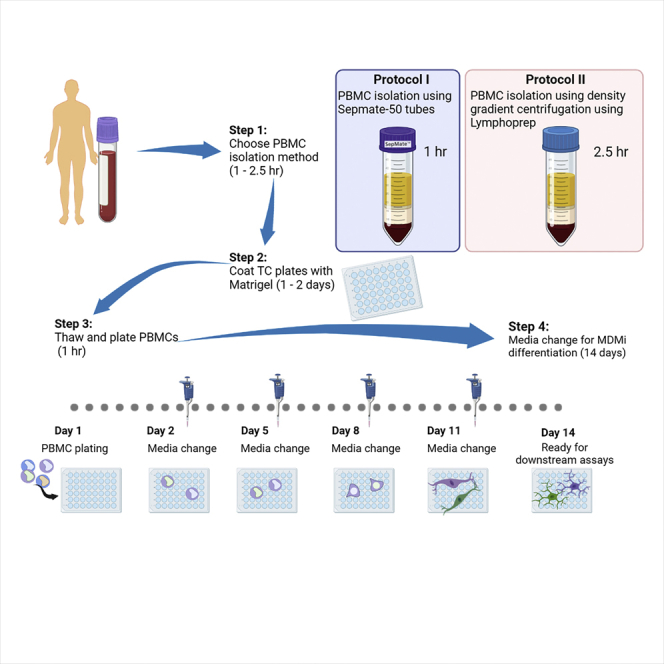

## Before you begin

1. Ensure you have received the appropriate institutional permission to work with human samples before proceeding with this protocol.
2. Prepare all solutions needed for monocyte-derived microglia-like cells (MDMi) differentiation as described below under materials and equipment.

### Institutional permissions (if applicable)

QIMR Berghofer Medical Research Institute’s Human Ethics Committee approved all the experimental protocols described below (P2197). This includes participant information and a consent form template for obtaining blood from healthy volunteers.

## Key resources table

**Table:** 

REAGENT or RESOURCE	SOURCE	IDENTIFIER
**Antibodies**		

P2RY12 (1:200)	Alomone Labs	Cat#APR-20, AB_11121048
Goat anti-Rabbit IgG (H+L) Highly Cross-Absorbed Secondary Antibody, Alexa Fluor 488 (1:250)	Thermo Fisher Scientific	Cat#A-11034, AB_2576217

**Biological samples**		

Human peripheral blood	In-house. Collected by a certified QIMRB institute phlebotomist. Healthy male and female volunteers, age range 20–60 years.	N/A

**Chemicals, peptides, and recombinant proteins**		

Granulocyte-macrophage-colony stimulating factor (GM-CSF)	PeproTech	Cat.#300-03
Recombinant Human IL-34	PeproTech	Cat.#200-34
Lymphoprep	STEMCELL Technologies	Cat.#07861
Fetal Bovine Serum, qualified, Australia (FBS)	Gibco	Cat.#10099141
Penicillin/streptomycin (100×)	Thermo Fisher Scientific	Cat.#15140122
RPMI 1640 Medium, GlutaMAX™ Supplement	Thermo Fisher Scientific	Cat.#61870036
Corning® Matrigel® Basement Membrane Matrix, LDEV-free, 10 mL	Bio-Strategy	Cat.#354234
DPBS (Ca^2+^Mg^2+^ free)	Thermo Fisher Scientific	Cat.#14190144
UltraPure™ 0.5 M EDTA, pH 8.0	Thermo Fisher Scientific	Cat.#15575020
Bovine Serum Albumin (BSA) fraction V (7.5%)	Thermo Fisher Scientific	Cat.#15260037
Paraformaldehyde (PFA)	Sigma	Cat.#P6148
Trypan Blue Solution, 0.4%	Gibco	Cat.#15250061
bisBenzimide H 33342 trihydrochloride (Hoechst 33342:1 μg /mL)	Sigma-Aldrich	Cat.#14533
Triton-X 100	Sigma-Aldrich	Cat.#X100

**Critical commercial assays**		

SepMate™-50 (IVD)	STEMCELL Technologies	Cat.#85460

**Software and algorithms**		

Fiji ImageJ	Fiji ImageJ	https://imagej.net/software/fiji/
Zeiss Zen Microscope software	ZEISS	https://www.zeiss.com/microscopy/int/products/microscope-software/zen.html

**Other**		

70 μm cell strainers	Corning	Cat.#352350
8-well chamber slides	DKSH Australia	Cat.#80821
48-well tissue culture plates	Sigma-Aldrich	Cat.#3548
9 mL Vacuette K3 EDTA	Interpath	Cat.#455036

## Materials and equipment

**Table:** PBMC wash solution

Reagent	Final concentration	Amount
DPBS	1×	499 mL
0.5 M EDTA	1 mM	1 mL
**Total**	**–**	**500 mL**

**Table:** Complete RPMI medium

Reagent	Final concentration	Amount
RPMI GlutaMAX	–	445 mL
100× pen/strep	1%	5 mL
FBS	10%	50 mL
**Total**	**–**	**500 mL**

**Table:** MDMi base medium

Reagent	Final concentration	Amount
RPMI GlutaMAX	–	495 mL
100× pen/strep	1%	5 mL
**Total**	**–**	**500 mL**

**Table:** PBMC cryopreservation medium

Reagent	Final concentration	Amount
FBS	90%	9 mL
DMSO	10%	1 mL
**Total**	**–**	**10 mL**

***Note:*** PBMC wash solution can be stored at RT for up to 1 month.

***Note:*** Complete RPMI medium can be stored at 4°C for up to 1 month or can be aliquoted and stored at −20°C.

***Note:*** MDMi base medium can be stored at 4°C for up to 1 month or can be aliquoted and stored at −20°C.

***Note:*** Always make up fresh PBMC cryopreservation medium. Do not store unused solution.

***Note:*** FBS is heat-inactivated at 56°C for 30 min as per the manufacturer’s instruction, aliquoted and stored in −20°C for long-term storage. Endotoxin levels for FBS is <0.50 EU/mL as per the Certificates of Analysis report.

***Note:*** IL-34 and GM-CSF are reconstituted in sterile water as per the manufacturer’s instruction (user would need to request a lot-specific datasheet from vendor). Add IL-34 (100 ng/mL) and GM-CSF (10 ng/mL) fresh to MDMi base medium before use. Do not prepare MDMi medium supplemented with growth factors for long-term storage.

***Note:*** Prepare PBMC cryopreservation medium fresh prior to freezing cells. Do not store left over cryopreservation medium.

**CRITICAL:** RPMI 1640 GlutaMAX should be stored in the dark at 2°C–8°C. FBS should be inactivated and stored at 4°C for short-term storage (1 week) and −20°C for long-term storage. Penicillin-streptomycin (pen/strep) should be stored at 4°C for short-term storage (1 week) and −20°C for long-term storage. All of these items need to be warmed to room temperature (23°C) before mixing.

**Table:** Immunocytochemistry antibody

Antibodies	Dilution factor (diluted in 2.5% BSA in PBS)	Final concentration
P2RY12	1:200	4 μg/mL
Goat anti-Rabbit IgG (H+L) Highly Cross-Absorbed Secondary Antibody, Alexa Fluor 488	1:250	2 μg/mL
bisBenzimide H 33342 trihydrochloride (Hoechst 33342)	1:1000	1 μg/mL

## Step-by-step method details

This protocol allows for the successful differentiation of MDMi for *in vitro* characterization. We will cover all steps that might affect MDMi differentiation ranging from the type of method (Protocol I vs Protocol II) for PBMC isolation, thawing of PBMCs, coating of culture plates and medium change for MDMi differentiation.

### PBMC isolation from whole blood

#### Protocol I: PBMC isolation using Sepmate-50^TM^ tubes

**Timing: 1 h**

This protocol enables the successful isolation of PBMCs for MDMi differentiation via the use of SepMate-50 tubes. This method reduces time and sample variability and improves yield and purity (https://www.stemcell.com/products/sepmate-50-ivd.html).

1. Collect 8–9 mL of human venous blood.
  a. A certified phlebotomist must perform blood collection.
  b. Any vacutainer blood tubes containing anti-coagulant can be used. For example: 9 mL Vacuette K3 EDTA tubes.
  c. Perform all future protocol steps within a tissue culture biohazard hood.

***Note:*** Blood should be stored at RT (not in fridge or 37°C/5% CO_2_ incubator) prior to processing. Process blood within 2 h from collection time. Decreased PBMCs recovery is expected for a longer blood processing time.

**Figure 1:**
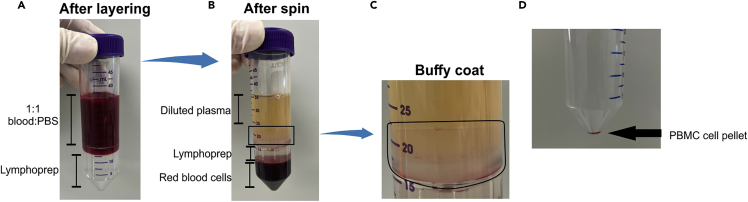
PBMC isolation using Sepmate-50^TM^ tube (A) Layer diluted blood with PBS into a Sepmate-50^TM^ tube containing Lymphoprep. (B) Diluted plasma, buffy coat, Lymphoprep and red blood cell layer after centrifugation. (C) The arrow indicates PBMCs being lodged on the side of the tube. (D) Presence of red blood cell contamination in PBMC cell pellet.
4. After removing the aliquot for cell counting, top up the 50 mL tube with PBS containing 1 mM EDTA and centrifuge 300 × *g* for 10 min at room temperature (23°C) with the brake on.
5. After centrifugation, remove the supernatant from the pellet.
6. PBMCs can be used after this step (see MDMi differentiation- step 21) or frozen down.
7. The PBMC cryopreservation medium is made up of 10% DMSO and 90% cold FBS (100 μL of DMSO and 900 μL of FBS).
  a. Dissolving DMSO in cold FBS will ensure that the cryopreservation medium does not heat up.
  b. Gently resuspend the PBMC pellet with 1 mL of cryopreservation medium and transfer to a pre-chilled cryovial labeled with the number of cells and date of freezing.
  c. Each cryovial should contain ∼1 × 10^7^ cells or per tube of blood (9 mL).
  d. Place the cryovial in a pre-chilled Nalgene® Mr. Frosty and store in −80°C freezer for 24 h.
  e. Transfer the vials to the vapor phase of liquid nitrogen after 24 h.

***Note:*** Do not re-use the unused cryopreservation medium.

***Note:*** Expected PBMC yields from whole blood (adult) ranges from 1.3–3 × 10^6^ cells/mL.

2. Use Sepmate-50^TM^ tube for PBMC isolation.
  a. Add 15 mL of Lymphoprep into the center hole of the Sepmate-50^TM^ tube as per the manufacturer’s instructions.
    ***Note:*** If bubbles are introduced, proceed to remove them by pipetting the bubble out.
  b. Gently mix the blood to ensure a homogeneous suspension.
  c. Diluted blood at 1:1 with room temperature (23°C) sterile PBS in a 50 mL Falcon tube.
    ***Note:*** Use PBS without Ca^2+^ and Mg^2+^.
  d. Gently pipette the diluted blood sample at the side of the Sepmate-50^TM^ tube.
  e. The blood sample should sit above the Lymphoprep (Figure 1A).
    ***Note:*** Use the lowest speed setting on the pipette gun, or use a pasture pipette.
    **CRITICAL:** Centrifuge the layered blood immediately.
  f. Centrifuge at 1,200 × *g* for 10 min at room temperature with the brake on at maximum.
  g. After centrifugation, different layers will be observed (from top to bottom: diluted plasma, buffy coat, Lymphoprep, red blood cell layer) (Figure 1B).
  h. If PBMCs appear to have adhered on the side of the Sepmate-50^TM^ tube, use a pasture pipette to dislodge the PBMCs carefully, without disrupting the layers. This will enhance PBMC cell recovery (Figure 1C).
  i. Transfer the top layer (buffy coat) containing PBMCs into a new 50 mL tube.
    ***Note:*** The buffy coat will look whitish.
  j. Top up the 50 mL tube with room temperature (23°C) sterile PBS containing 1 mM EDTA to prevent cell clumps and resuspend the cell pellet.
  k. Centrifuge at 300 × *g* for 10 min at room temperature (23°C) with the brake on at maximum.
  l. After centrifugation, aspirate the supernatant from the pellet and gently resuspend the cells in 1 mL of PBS containing 1 mM EDTA.
    ***Note:*** There will be some visible red blood cell contamination, which will be lost after cryopreservation and downstream assays (Figure 1D).
    ***Note:*** Minimize the time that the PBMCs remain in pellet.
3. Perform a cell count by removing 10 μL for a Trypan Blue exclusion using a hemocytometer (Strober 2001) or an automated cell counter.

**CRITICAL:** FBS must be heat-inactivated prior to use (see materials and equipment).

#### Protocol II: PBMC isolation using density gradient centrifugation using Lymphoprep

**Timing: 2.5 h**

This protocol enables the successful isolation of PBMCs for MDMi differentiation using density gradient centrifugation.

8. Collect 8–9 mLs of human venous blood.
  a. A certified phlebotomist must perform blood collection.
  b. Any vacutainer blood tubes containing anti-coagulant can be used. For example: 9 mL Vacuette K3 EDTA tubes.
  c. Perform all future protocol steps within a tissue culture biohazard hood.
9. Gently mix the blood to ensure a homogeneous suspension.
  a. Dilute blood at 1:1 with room temperature (23°C) sterile PBS in a 50 mL Falcon tube.

***Note:*** Use PBS without Ca^2+^ and Mg^2+^.

10. Prepare density gradient solution in a 50 mL tube for PBMC isolation.
  a. Add 7.5 mL of room temperature (23°C) Lymphoprep to a 50 mL tube.
  b. Carefully layer the diluted blood on top of the Lymphoprep using a transfer pipette.
  c. Slowly drip the diluted blood against the side of the 50 mL tube to avoid mixing with the Lymphoprep.
  d. Centrifuge at 400 × *g* for 30 min at room temperature (23°C), **NO BRAKE** (1/10) for deceleration to prevent red blood cells remixing with PBMCs.
    ***Note:*** Centrifuge blood immediately after layering. Blood will gradually mix with Lymphoprep if not centrifuged. The tube can be spun for an additional 10 min with the break-off.
    **CRITICAL:** Leaving the break on will disrupt the layers.
  e. Gently transfer the buffy coat to a new 50 mL tube with a transfer pipette.
    ***Note:*** Avoid getting any of the Lymphoprep and red blood cell layer.
  f. Top up 50 mL tube with PBS containing 1 mM EDTA and centrifuge at 300 × *g* for 10 min. Brake can be turned on.
  g. After centrifugation, aspirate the supernatant from the pellet and gently resuspend the cells in 1 mL of PBS with 1 mM EDTA.
    ***Note:*** There will be some visible red blood cell contamination, which will be lost after cryopreservation and downstream assays.
11. Perform a cell count by removing 10 μL for a Trypan Blue exclusion using a hemocytometer or automated cell counter.
12. After removing the aliquot for cell counting, top-up 50 mL with PBS containing 1 mM EDTA and centrifuge 300 × *g* for 10 min.
13. To freeze down PBMCs- see Protocol 1 – step 7.

**CRITICAL:** All steps of PBMC isolation must be completed using room temperature (23°C) buffers.

***Alternatives:*** Lymphoprep can be substituted for Ficoll-Paque Plus (GE healthcare Pharmacia- 17-1440-03). These products have a density of 1.077 g/mL suitable for PBMC isolation.

### Coating procedure using corning Matrigel basement matrix

**Timing: 1–2 day**

This protocol ensures proper coating of tissue culture plates using Matrigel for successful differentiation of MDMi (https://www.corning.com/catalog/cls/documents/faqs/CLS-DL-CC-026.pdf).

14. Thaw Matrigel basement membrane matrix overnight (16–24 h).
  a. Thaw Matrigel by submerging the vial in ice at 4°C overnight (16–24 h) as per the manufacturer’s recommendation.
  b. The next day, swirl the Matrigel vial to ensure homogeneity before use.
    ***Note:*** Do not freeze/thaw Matrigel. Aliquot and store Matrigel at −80°C for future use.
    **CRITICAL:** Matrigel basement membrane matrix is temperature sensitive and will solidify (gelled) above 10°C. Keep Matrigel on the ice at all times and use pre-chilled disposables.
  c. Prepare ice-cold sterile PBS and chill pipette tips in ice-cold PBS by pipetting repeatedly or in the fridge for 10 min to prevent Matrigel gelling on pipette tip causing inaccuracy.
  d. Spray down the ice bucket containing Matrigel with 70% v/v ethanol before placing it in the tissue culture hood.
  e. Perform all subsequent procedure within a tissue culture hood.
15. Thinly coat tissue culture plates with 100 μg/mL of Matrigel diluted in ice-cold PBS.
  a. Cover the surface of the tissue culture plates. For example, add 200 μL /cm^2^ of Matrigel coating solution to the growth surface.
  b. Resuspend solution until Matrigel is dissolved in PBS (herein referred as Matrigel coating solution).
    ***Note:*** If Matrigel has gelled in PBS, leave the solution in the fridge for 10 min until it dissolves.
  c. Ensure that the plate’s total surface area is covered with the Matrigel coating solution.
16. Place plates in a 37°C/5% CO_2_ incubator overnight (16–24 h).

***Note:*** Matrigel's protein concentration ranges between 8-12 mg/mL depending on the lot number. Check the concentration of Matrigel basement membrane matrix: https://www.corning.com/worldwide/en/products/life-sciences/resource-library.html?productNumber=356230&lotNumber=.

***Alternatives:*** Matrigel can be substituted with Geltrex matrix (Thermo Fisher Scientific - Cat#A1413202).

**CRITICAL:** Do not coat plates with undissolved Matrigel. Do not store unused Matrigel at 4°C post-thaw/dilution. Instead, aliquot Matrigel and store at −20°C.

***Note:*** Matrigel-coated plates are best used the same day, but can be stored in the incubator at 37°C for up to a week in Matrigel coating solution. Do not allow the coated surface to dry out.

### Establish PBMC cultures for MDMi differentiation

This protocol allows for the successful differentiation of MDMi for *in vitro* characterization. We will cover all steps that might affect MDMi differentiation from thawing PBMCs to coating culture plates and medium changes.

### PBMC thawing protocol to optimize cell recovery for MDMi differentiation

**Timing: 1 h**

This protocol is optimized for maximum PBMC recovery after thawing for MDMi differentiation.

17. Prepare pre-warmed complete RPMI medium in a water bath.
  a. Ensure that the FBS is heat-inactivated.
18. Thaw PBMC cryovial at 37°C in a water bath by gentle shaking the vial, and remove when a small amount of ice crystal remains.
  a. Wipe down PBMC cryovial with 70% v/v ethanol before transferring into the tissue culture hood.
  b. Transfer PBMC from the cryovial to a 10 mL tube.
  c. Drop-wise addition of pre-warmed complete RPMI medium into the tube containing PBMCs at a rate of 1 drop per second while swirling the sample until 4 mL. Top up to 10 mL with pre-warmed complete RPMI medium.
    ***Note:*** Drop-wise addition of complete RPMI medium is critical to minimize osmotic shock to ensure maximum viability of the cells.
    **CRITICAL:** All thawing steps must be completed using pre-warmed medium for best cell viability and recovery.
  d. Centrifuge sample at 300 × *g* for 5 min at room temperature (23°C).
  e. Aspirate the supernatant and resuspend the cells in 1 mL of pre-warmed complete RPMI medium.
19. Perform a cell count by removing 10 μL for a Trypan Blue exclusion using a hemocytometer or automated cell counter.

***Note:*** If PBMCs appear clumpy, use a 70 μm cell strainer to remove clumps to obtain a single cell suspension.

20. Proceed with the MDMi differentiation step.

### MDMi differentiation

**Timing: 14 days**

**Figure 2:**
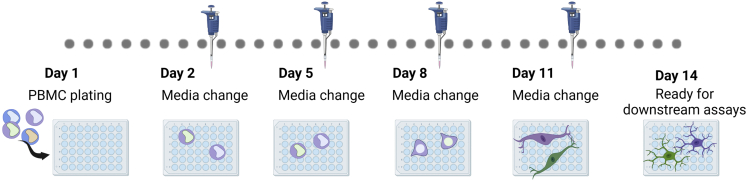
Timeline for medium changes across 14 days of MDMi differentiation

This protocol describes the differentiation of MDMi from PBMCs. MDMi were differentiated for 14 days prior to downstream assay. We have also outlined a timeline for medium changes across 14 days of MDMi differentiation (Figure 2).

**Figure 3:**
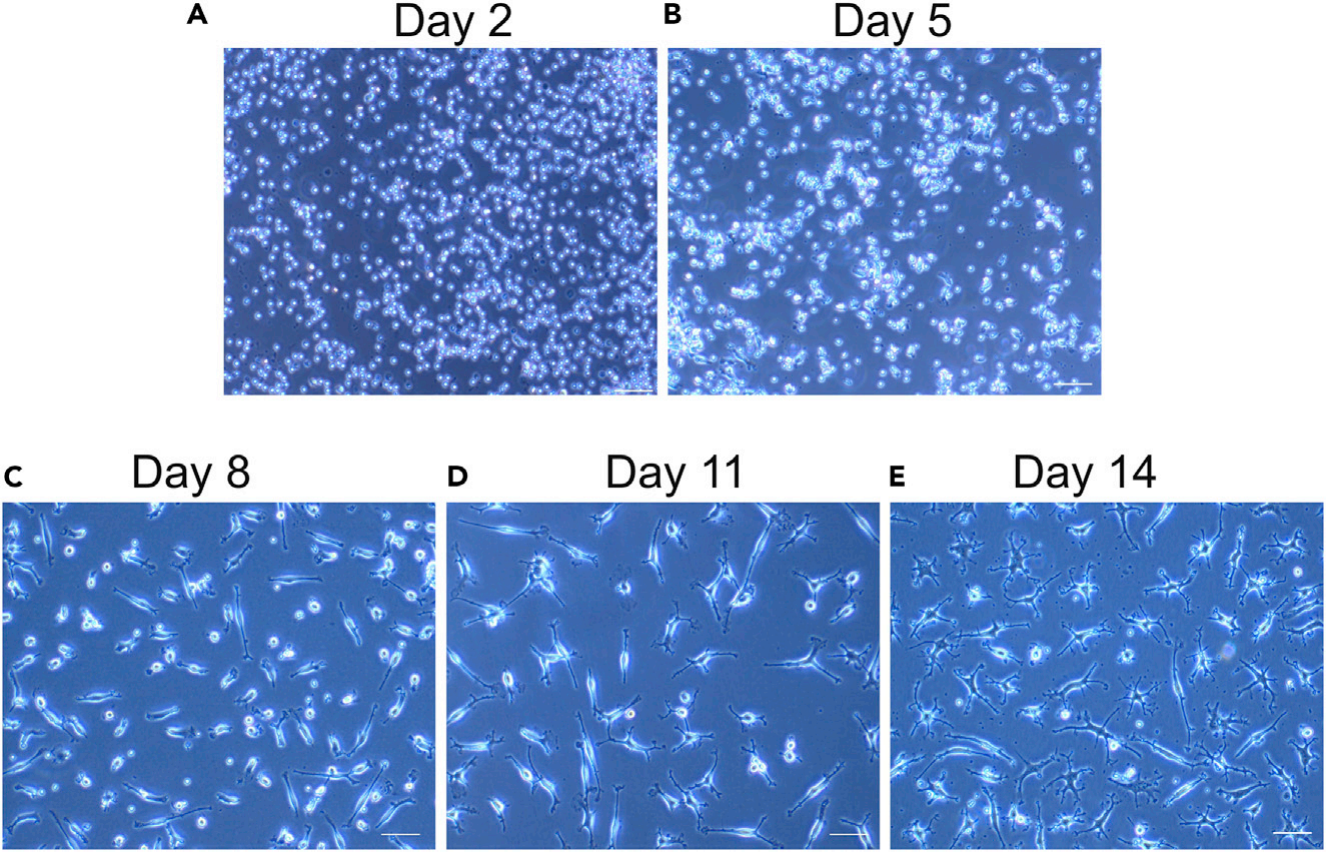
Representative images of MDMi throughout 14 days of differentiation (A) Mixed culture of monocyte and lymphocytes on Day 2. (B) Monocytes adhering to the plate’s bottom appear irregular in shape while lymphocytes remains rounded at Day 5. (C) The elongated appearance of monocytes on Day 8. (D) Cells showed an increase in ramification on Day 11. (E) Fully ramified MDMi on Day 14. Scale bar, 50 μm.
  e. MDMi can be used on day 14 for downstream experiments.

***Note:*** MDMi are non-proliferative and will lose its morphology if re-plated. Therefore the user needs to determine the required cell number prior to plating, in accordance to the assay that will be performed.

21. Differentiate MDMi in a 37°C/5% CO_2_ incubator for 14 days, as follows:
  a. On day 1, plate 4 × 10^5^ PBMCs/cm^2^ in complete RPMI medium.
  b. Oscillate the plate gently to disperse the cells homogeneously across the plate (day 1), and leave the plate for 5 min before putting them in the incubator.
  c. Leave plate overnight (16–24 h) in a 37°C/5% CO_2_ incubator.
  d. On day 2 in culture, remove all medium from the plate and replace with fresh MDMi base medium containing 100 ng/mL IL-34 and 10 ng/mL GM-CSF.
    i. Perform a medium change every 3 days (day 5, day 8, and day 11) by removing half the medium and replacing it with fresh MDMi base medium containing 2× cytokines (i.e., 200 ng/mL IL-34 and 20 ng/mL GM-CSF (Figure 3).

### Characterization of MDMi by immunocytochemistry

**Timing: 15 days**

**Figure 4:**
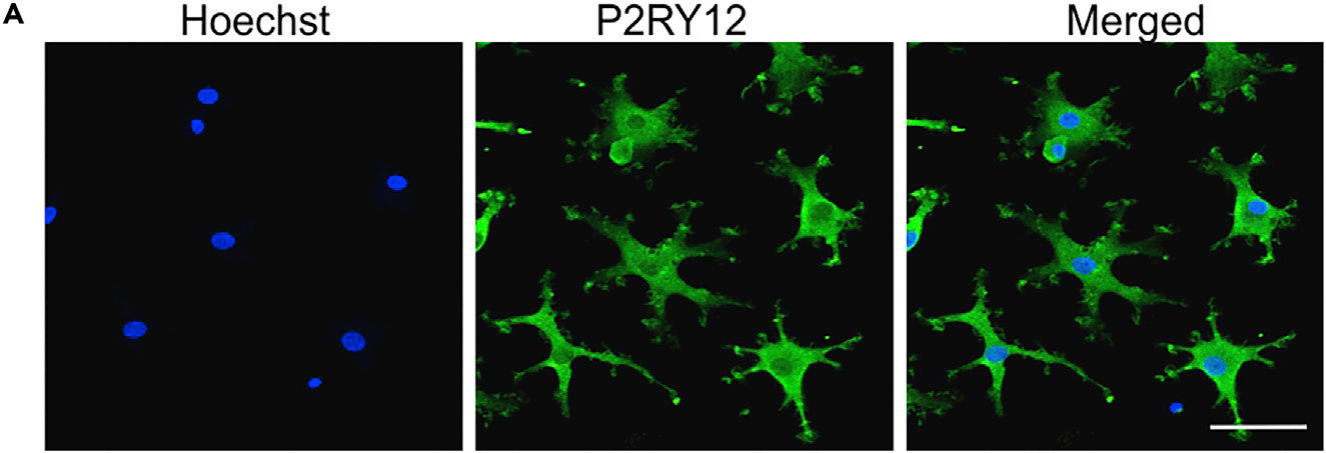
Immunocytochemistry of day 14 MDMi (A) Representative image of day 14 MDMi stained for P2RY12. Scale bar, 50 μm.

This protocol describes how to prepare MDMi for analysis by immunocytochemistry with a microglia-specific marker. MDMi were differentiated for 14 days and stained for P2RY12 (Figure 4).

22. Prepare imaging chamber slides.
  a. Coat 8-well chamber slides with 400 μL of 100 μg/mL of Matrigel in cold PBS by incubating overnight (16–24 h) at 37°C. Remove Matrigel solution and rinse each chamber three times with PBS before plating PBMCs.
23. Plate PBMCs and differentiate for 14 days as follows:
  a. Plate 3 × 10^5^ /400 μL PBMCs on Matrigel-coated chamber slides and culture in a 37°C/5% CO_2_ incubator.
  b. Change medium every third day until Day 14 (see MDMi differentiation – step 21).
24. Aspirate the cell culture medium and wash cells once with PBS.
25. Fix cells with 400 μL (per well) of 4% paraformaldehyde (PFA) in PBS for 15 min at RT. Wash coverslips thrice with PBS.
26. For permeabilization, incubate cells with 400 μL (per well) of 0.3% Triton-X 100 in 2.5% BSA in PBS for 10 min at RT. Cells do not need to be wash after this step.
27. Block non-specific binding with 400 μL (per well) of 2.5% BSA in PBS for 1 h at RT.
28. Replace blocking solution with 400 μL (per well) of primary antibody diluted in 2.5% BSA in PBS (see materials and equipment for antibody details) for 1 h at room temperature (23°C).
29. Wash chamber slides 5 times with 500 μL (per well) of PBS for 5 min each.
30. Replace PBS with 400 μL (per well) secondary antibody with 1 μg/mL Hoechst 33342 (nuclear dye) diluted in 2.5% BSA in PBS (see materials and equipment for antibody details) for 2 h, in the dark at room temperature (23°C).
31. Wash chamber slides 5 times with 500 μL (per well) of PBS for 5 min each.
32. Add 500 μL (per well) of PBS. Slides can be imaged immediately or stored at 4°C for long-term storage.

***Note:*** Samples were visualized on a Zeiss LSM-780 NLO with ZEN software. Image analysis was performed with Image J. Background signal and secondary only control were used to subtract from images in FIJI software using the function “Process> Subtract background”.

## Expected outcomes

The protocol provides a consistent and robust method to differentiate MDMi from healthy individuals and patients with various neurodegenerative diseases. MDMi differentiated for 14 days will show a small soma and complex branching structures that resemble brain microglia (Figure 3). These cells can be further processed for various assays, including phagocytosis, ELISA, RT-qPCR, flow cytometry, western blot and immunocytochemistry, and can be further co-cultured with other brain cell types (Carla et al., 2021). The number of MDMi cells that can be differentiated depends on the number of monocytes per given volume in an individual’s blood. MDMi can be utilized to investigate the molecular and biochemical mechanisms related to immune-related and neurodegenerative diseases, and can be used to study patient and disease heterogeneity to provide a basis for personalized medicine.

## Limitations

This protocol has been successfully used to differentiate MDMi from over 100 healthy and patient blood samples. The failure of MDMi differentiation can result from poor PBMC isolation and cryopreservation, leading to poor cell numbers and viability. MDMi are non-proliferative, therefore, the total number of PBMCs needs to be determined before plating, considering the number of downstream assays that needs to be performed.

PBMC isolation using SepMate-50 tubes (Protocol I) increases cell yield and consistency and reduces processing time, making it a favorable isolation method compared to Protocol II, which involves the traditional layering of blood via density gradient separation. Additionally, workflow using SepMate-50 tubes for PBMC isolation can be standardized across multiple sites, reducing user variability. While SepMate-50 tubes are costly and require twice the amount of Lymphoprep (15 mL) compared to Protocol II (7.5 mL) for a single tube of blood, it significantly reduces the length of processing time and increases the consistency in PBMC isolation which are both critical for efficient MDMi differentiation. Despite the differences between PBMC yields when using Protocol I or Protocol II, MDMi generated using either of these protocols showed similar cell morphology and microglia marker expression.

## Troubleshooting

### Problem 1

Do not have SepMate-50^TM^ tube tubes for PBMC isolation (Protocol I).

### Potential solution

Use Protocol II of PBMC isolation as an alternative method for successful MDMi differentiation. Protocol II has a longer processing time and generates a lower yield of PBMCs (2%–5% lesser than Protocol I) but these factors do not affect MDMi differentiation. Previous studies have shown similar PBMC yields using Ficoll vs Sepmate tubes (Betsou et al., 2019).

### Problem 2

Poor PBMC yield due to cell clumping. (step 18).

### Potential solution

PBMC clumps will underestimate cell density for differentiation which will impede quality of cell, morphology and maturation of MDMi.

There are several potential solutions to this problem:

• Filter PBMCs through a 70 μm cell strainer to obtain a single cell suspension.
• If cell clumps are still visible, DNAse I at 200 U/mL can be added in pre-warmed medium (in 9 mL) when thawing cells. DNAse I treated PBMCs can be incubated for 20 min at 37°C and then centrifuged for 300 × *g* for 5 min before plating.
• Use room temperature (23°C) buffers when isolating PBMCs.

### Problem 3

MDMi display an amoeboid morphology and/or are not ramified.

### Potential solution

MDMi cultures are sensitive to culture conditions, and may display an amoeboid morphology indicative of cell activation.

There are several potential solutions to this problem:

• At the first indication of an amoeboid morphology, consider removing existing medium and rinse thrice with MDMi base medium. Replace with fresh MDMi base medium supplemented with 100 ng/mL IL-34 and 10 ng/mL GM-CSF and leave cultures for 2–3 more days. If problem persists, consider restarting cultures.
• If amoeboid MDMi are observed after 10 days of differentiation, consider restarting cultures immediately.
• Cell density affects MDMi differentiation and ramification. Consider optimizing PBMC counts prior to MDMi differentiation.
• The type of culture surface also impedes MDMi differentiation. We find that MDMi differentiated in glass slides are less ramified. It is recommended to use polymer slides.
• Perform batch testing of FBS for low endotoxin or check if FBS was inactivated appropriately as per the manufacturer’s instruction.

### Problem 4

Is there an alternative to Matrigel? (step 14).

### Potential solution

Coating using Geltrex matrix has shown to be capable of differentiating MDMi (Sellgren et al., 2019).

### Problem 5

Gaps in the culture were observed after medium change. (step 21).

### Potential solution

There are several potential solutions to this problem:

• Be gentle with medium changes to prevent cell loss (i.e., pipette against the wall of the well).
• Tissue culture plates may not be coated well. Ensure that Matrigel is completely dissolved in cold PBS before coating.

### Problem 6

There seem to be a large number of unbound cells (lymphocytes) in culture.

### Potential solution

The number of lymphocytes will decrease after each media change. Wells should be washed thoroughly with MDMi base medium before proceeding with downstream experiments.

### Problem 7

EDTA blood collection tubes are not available. Can we use other anti-coagulant blood tubes for PBMC isolation? (step 1).

### Potential solution

Other anti-coagulant blood collection tubes are available for PBMC isolation (i.e., heparin tubes). We have not trialed other blood tubes to isolate PBMC, other than K3 EDTA. However, a previous study has shown an increase in CD14^+^ monocytes in PBMC isolation in EDTA compared to heparin tube (Rundgren et al., 2018).

## Resource availability

### Lead contact

Further information and requests should be directed to and will be fulfilled by the lead contact, Anthony R. White (Tony.white@qimrberghofer.edu.au).

### Materials availability

This study did not generate new unique reagents.

## Data Availability

This study did not generate/analyze datasets/code.
